# Optimizing cardiopulmonary bypass management beyond duration: insights from the sequential organ failure assessment score after cardiac surgery

**DOI:** 10.1093/icvts/ivae153

**Published:** 2024-09-03

**Authors:** Ignazio Condello

**Affiliations:** Department of Cardiac Surgery, GVM Care & Research, Anthea Hospital, Bari, Italy

**Keywords:** Cardiopulmonary bypass, Management, Duration, Sequential Organ Failure Assessment Score, Cardiac surgery

Dear Editor,

I am writing to discuss the article ‘The impact of cardiopulmonary bypass time on the Sequential Organ Failure Assessment score after cardiac surgery’ by Tiago R. Velho *et al.*, published in your esteemed journal. This retrospective study provides important insights into the association between cardiopulmonary bypass (CPB) duration and postoperative organ dysfunction, as measured by the Sequential Organ Failure Assessment (SOFA) score [1]. While the study clearly demonstrates that prolonged CPB times correlate with higher SOFA scores and increased morbidity and mortality risks, it also brings to light that CPB duration alone is not the sole determinant of patient outcomes. Several other critical factors in CPB management require attention to minimize postoperative complications. Effective venous return is essential for maintaining adequate perfusion and preventing venous air embolism. Inadequate venous return can compromise cardiac output, leading to organ dysfunction. Future research should explore enhanced venous return techniques, such as vacuum-assisted venous drainage and optimized cannulation strategies, to improve postoperative outcomes [2]. Maintaining optimal haemoglobin levels during CPB is critical for ensuring sufficient oxygen delivery to tissues. Anaemia during CPB can exacerbate tissue hypoxia and contribute to organ dysfunction. Implementing intraoperative haemoglobin management protocols, including ultrafiltration and blood conservation strategies, could significantly reduce postoperative SOFA scores. Temperature regulation during CPB is vital to prevent complications associated with hypothermia and hyperthermia. Both extremes can adversely affect organ function. Investigating advanced temperature management techniques, such as goal-directed normothermia or controlled hypothermia, and their influence on SOFA scores could provide valuable insights into improving patient outcomes. Haemolysis, caused by mechanical stress on red blood cells during CPB, leads to the release of free haemoglobin with pro-inflammatory and oxidative properties. Reducing haemolysis through biocompatible materials and optimized pump flow rates is crucial [3]. Exploring the relationship between haemolysis markers and SOFA scores could inform better CPB practices. The biocompatibility of materials used in CPB circuits, including tubing and oxygenators, significantly impacts the inflammatory response and complement activation. Innovations in biocompatible materials that minimize these adverse effects could reduce the incidence of postoperative organ dysfunction. Comparative studies focussing on different CPB materials and their impact on SOFA scores would be highly beneficial. In conclusion, while Velho *et al.*’s study provides valuable insights into the impact of CPB duration on postoperative organ dysfunction, it also underscores that duration is not the only critical factor. Effective CPB management requires a comprehensive approach that includes optimizing venous return, maintaining appropriate haemoglobin levels, regulating temperature, preventing haemolysis and utilizing biocompatible materials. By addressing these factors, we can enhance patient outcomes and reduce the incidence of postoperative complications.
